# Association between red blood cell distribution width-to-albumin ratio and prognosis in post-cardiac arrest patients: data from the MIMIC-IV database

**DOI:** 10.3389/fcvm.2024.1499324

**Published:** 2025-01-07

**Authors:** Yinhe Cai, Yao Zhang, Ningzhi Zhou, Yong Tang, Haixia Zheng, Hong Liu, Jiahua Liang, Ruixiang Zeng, Shengqing Song, Yu Xia

**Affiliations:** ^1^ Department of Cardiology, The Third Affiliated Hospital of Guangzhou University of Chinese Medicine, Guangzhou, China; ^2^ Guangzhou University of Chinese Medicine, Guangzhou, China; ^3^ The Department of Cardiovascular Disease, Meizhou Hospital of Traditional Chinese Medicine, Meizhou, China; ^4^ Department of Critical Care Medicine, Guangdong Provincial Hospital of Chinese Medicine, Guangzhou, China; ^5^ The Third Affiliated Hospital of Guangzhou University of Chinese Medicine, Guangzhou, China

**Keywords:** all-cause mortality, cardiac arrest, red blood cell distribution width, albumin, MIMIC-IV database

## Abstract

**Background:**

Cardiac arrest (CA) triggers a systemic inflammatory response, resulting in brain and cardiovascular dysfunction. The red blood cell distribution width (RDW)-to-albumin ratio (RAR) has been widely explored in various inflammation-related diseases. However, the predictive value of RAR for the prognosis of CA remains unclear. We aimed to explore the correlation between the RAR index and the 30- and 180-day mortality risks in post-CA patients.

**Methods:**

Clinical data were extracted from the MIMIC-IV database. The enrolled patients were divided into three tertiles based on their RAR levels (<3.7, 3.7–4.5, >4.5). Restricted cubic spline, Kaplan–Meier (K-M) survival curves, and Cox proportional hazards regression model were used to explicate the relationship between the RAR index and all-cause mortality risk. Subgroup analyses were also conducted to increase stability and reliability. The receiver operator characteristic (ROC) analysis was used to assess the predictive ability of the RAR index, red blood cell distribution width, and serum albumin for 180-day all-cause mortality.

**Results:**

A total of 612 patients were eligible, including 390 men, with a mean age of 64.1 years. A non-linear relationship was observed between the RAR index and 180-day all-cause mortality, with a hazards ratio (HR) >1 when the RAR level exceeded 4.54. The K-M survival curve preliminarily indicated that patients in higher tertiles (T2 and T3) of the RAR index presented lower 30- and 180-day survival rates. An elevated RAR index was significantly associated with an increased 30-day [adjusted HR: 1.08, 95% confidence interval (CI): 1.01–1.15] and 180-day (adjusted HR: 1.09, 95% CI: 1.03–1.16) mortality risk. According to the ROC curve analysis, the RAR index outperformed the RDW and albumin in predicting all-cause 180-day mortality [0.6404 (0.5958–0.6850) vs. 0.6226 (0.5774–0.6679) vs. 0.3841 (0.3390–0.4291)]. The prognostic value of the RAR index for 180-day mortality was consistent across subgroups, and a significant interaction was observed in patients who were white, those with chronic pulmonary disease, or those without cerebrovascular disease.

**Conclusion:**

The RAR index is an independent risk factor for 30- and 180-day all-cause mortality in post-CA patients. The higher the RAR index, the higher the mortality. An elevated RAR index may be positively associated with adverse prognosis in post-CA patients, which can remind clinicians to quickly assess these patients.

## Introduction

Cardiac arrest (CA) is one of the leading fatal diseases, characterized by the sudden cessation of cardiac ejection due to various causes (1). Only 25% of patients survive after experiencing CA, and more than 300,000 inpatients suffer from CA each year in the United States (2). Hence, early and accurate prediction associated with death in CA patients is particularly significant, contributing to stratifying high-risk patients swiftly and helping clinicians take more appropriate treatments in clinical practice.

Red blood cell distribution width (RDW) is a commonly used indicator in routine clinical hematology. Existing evidence indicates that RDW has been a novel and easily convenient marker of systemic inflammation. It has been reported that higher RDW levels are associated with worse prognoses in cardiovascular diseases (3, 4), ischemic stroke (5), kidney disease (6), and various infectious diseases (7). However, the RDW level is known to be unstable, as it is susceptible to other factors such as senescence, oxidative stress, malnutrition, or renal impairment (8). Furthermore, serum albumin (ALB) is an indicator of systemic nutritional status, and it can reduce the systemic inflammatory response by inhibiting oxidative stress and accelerating endothelial apoptosis (9, 10). Previous studies have identified low levels of serum albumin as an independent risk factor for increased cardiovascular mortality (11, 12). However, serum albumin levels are also susceptible to other factors such as chronic diseases, nutritional risk, malnutrition, and inflammation (13). Several studies have suggested that the combined marker—RDW-to-albumin ratio (RAR)—is a novel biomarker associated with the prognosis of circulation system diseases, such as stroke (9), acute myocardial infarction (14), and atrial fibrillation (15). Compared to other inflammatory markers, the RAR index being rapid, reproducible, and easy to acquire means it could be used for routine screening in clinical practice through laboratory testing. The RAR index serves as a composite marker that combines both nutritional status (ALB) and inflammatory status (RDW), and it reflects a better correlation with the inflammatory response and identification of high-risk patients compared to other single-identified markers. It has been reported that post-CA patients with high levels of inflammatory response face higher mortality rates and/or worse neurologic consequences (16, 17). However, whether the RAR index can more accurately assess the prognosis of post-CA patients has not yet been evaluated.

In this study, we aimed to explore the correlation between the RAR index and 30- and 180-day mortality in post-CA patients enrolled in the intensive care unit (ICU).

## Materials and methods

### Sources of data

All data were collected from the Medical Information Mart for Intensive Care IV (MIMIC-IV; version 2.0) database, a large and open critical care database approved by the Beth Israel Deaconess Medical Center and the Massachusetts Institute of Technology. This database contains records from 76,540 ICU patients admitted between 2008 and 2019. The longest follow-up period for each patient in the database was 1 year after their last discharge, providing available data support for clinical research. The personal information of all included patients was de-identified to ensure privacy of patients. Therefore, the informed consent of patients was exempt for this study.

### Sample size and power analysis

The sample size was predetermined due to the retrospective design of the study (18). A *post-hoc* power analysis was performed using PASS 24.0 software to assess the study's statistical power, which was calculated to exceed 90%.

### Study population

Patients who met the following criteria were enrolled: (1) patients diagnosed with CA based on the International Classification of Diseases versions 9 and 10 (ICD-9 and 10) diagnosis codes (“4,275,” “I46,” “I,462,” “I,468,” and “I,469”); (2) only the first ICU admission during the initial hospitalization was considered; and (3) age 18 years and above. Exclusion criteria were as follows: (1) patients without recorded RDW or albumin levels; and (2) ICU length of stay <24 h.

### Data extraction

Data were extracted from the MIMIC-IV database based on three parts: (1) baseline characteristics, including sex, age, ethnicity [white, or others (Asian, Black, unknown)], weight, comorbidities (cardiovascular disease, congestive heart failure, chronic lung disease, diabetes, peripheral vascular disease, and cerebrovascular disease), the Sequential Organ Failure Assessment (SOFA) score, the Simplified Acute Physiology Score II (SAPSII), and norepinephrine use; (2) vital signs, including systolic blood pressure (SBP), diastolic blood pressure (DBP), heart rate, respiratory rate, and pulse oxygen saturation (SpO_2_); and (3) laboratory biomarkers, including RDW, red blood cell (RBC) count, white blood cell (WBC) count, platelet (PLT) count, hemoglobin, albumin, creatinine, blood urea nitrogen (BUN), glucose, international normalized ratio (INR), anion gap, alanine aminotransferase (ALT), aspartate aminotransferase (AST), alkaline phosphatase (ALP), total sodium, and potassium. All data were extracted within the first 24 h after admission to the ICU, excluding variables with ≥10% missing values. For continuous variables with less than 5% missing data, we imputed with the median of non-missing values. Variables with 5%–10% missing data were filled with multiple imputations. Data were obtained using Navicat Premium version 15.0.

### Groups and outcomes

The exposure factor, RAR index, was calculated as RDW (%) divided by albumin (g/dl) (19). Violin plots and histogram density plots were prepared to visualize the fundamental characteristics of the RAR index. A total of 612 patients were enrolled in our study and divided into three groups based on the tertiles of the RAR index (20): T1 (<3.7, *n* = 139), T2 (3.7–4.5, *n* = 150), and T3 (>4.5, *n* = 323).

The endpoints of this study were 30- and 180-day all-cause mortality.

### Statistical analysis

Continuous data that conformed to normal distribution are presented as the mean ± standard deviation (SD) and were analyzed using one-way ANOVA; data with a skewed distribution are presented as the median and quartile spacing [M (Q1, Q3)] and were analyzed using the Kruskal–Wallis test. Categorical variables are presented as *n* (%), with the analysis of the chi-square test (or Fisher's exact test).

The cumulative probability of 30- and 180-day all-cause mortality was visualized across the RAR index tertiles (T1, T2, T3) using Kaplan‒Meier (K‒M) curves with the log-rank test. Univariate and multivariate Cox proportional hazards regression models were used to evaluate the prognostic values of the RAR index for 30- and 180-day all-cause mortality, and hazards ratios (HRs) and 95% confidence intervals (CIs) were presented. Model 1 was adjusted for age, sex, weight, and race. Model 2 was adjusted for age, sex, weight, race, SOFA score, SAPSII score, cardiovascular disease, chronic pulmonary disease, diabetes, cerebrovascular disease, peripheral vascular disease, norepinephrine use, creatinine, SBP, DBP, ALT, ALP, and AST. In addition, subgroup analyses were also conducted based on different strata, such as demographic characteristics (sex, age, and race), comorbidities (cardiovascular disease, chronic pulmonary disease, diabetes, cerebrovascular disease, and peripheral vascular disease), and norepinephrine use.

Restricted cubic spline (RCS) analysis, based on the Cox model, was utilized to elucidate the dose–response relationship between the RAR index and 180-day all-cause mortality in post-CA patients. Receiver operating characteristic (ROC) analysis was performed to predict 180-day mortality in CA patients among RDW, albumin, RAR index, SOFA score, and SAPSII score. The above statistical analyses were performed using Stata 15.0 and SPSS 23.0 software. *P-*values <0.05 were considered statistically significant.

## Results

### Clinical characteristics of patients

**Figure 1:**
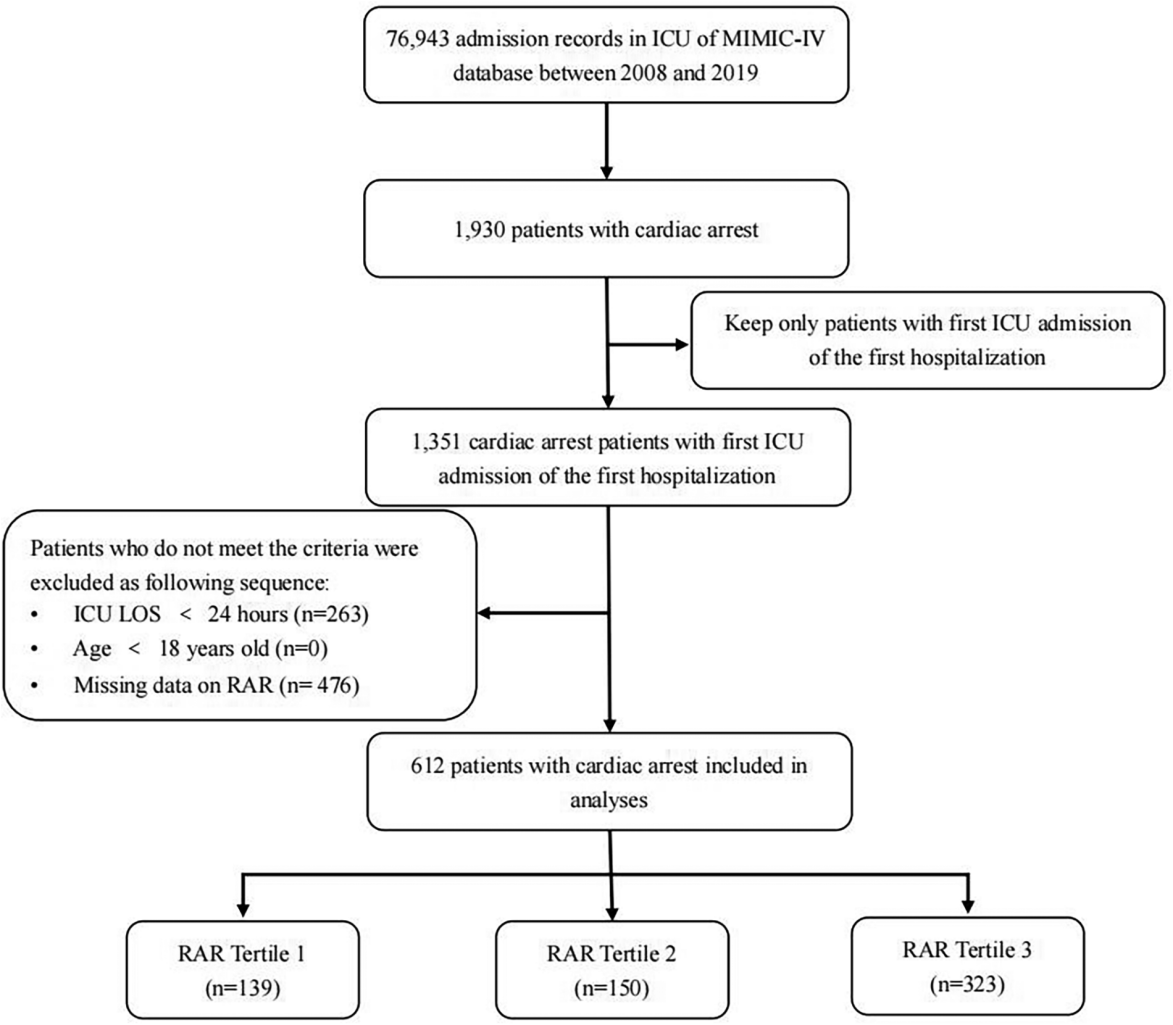
Flowchart of patient screening.

**Figure 2:**
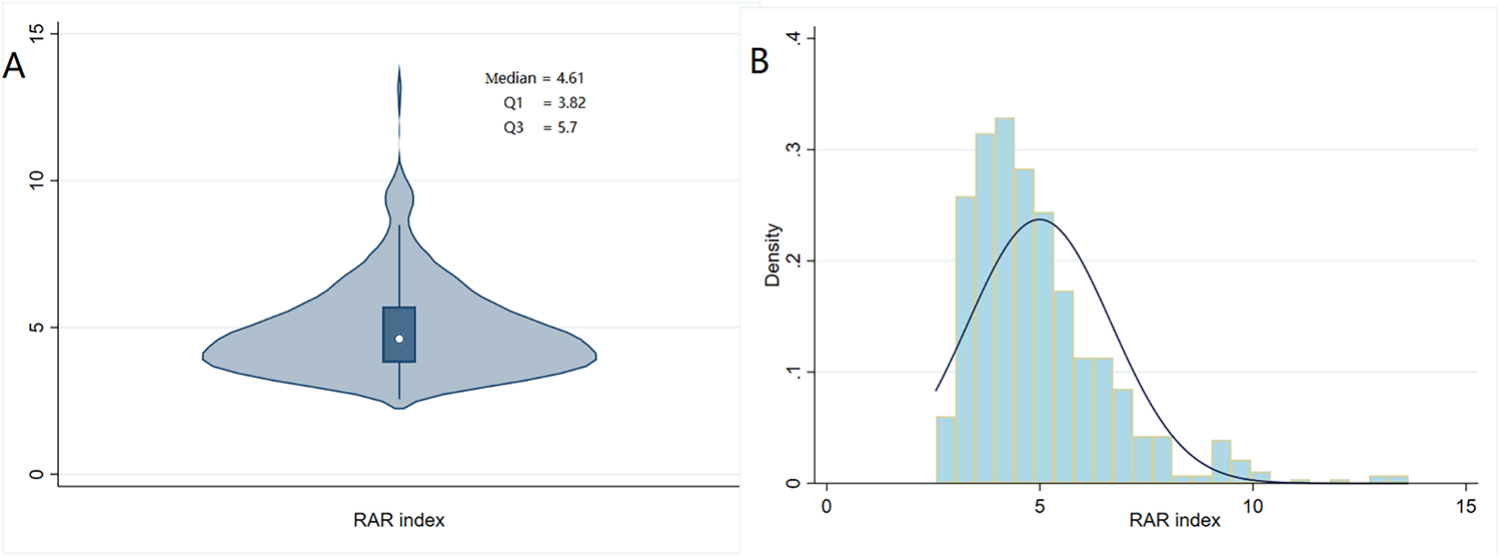
Violin plot (**A**) and histogram density plot (**B**) displaying the distribution of the RAR index.

We included data of 612 eligible CA patients from the MIMIC-IV database (see Figure 1). In general, 318 patients (51.96%) and 362 patients (59.15%) died of various causes within 30 and 180 days after admission. According to Table 1, the average age of the included patients was 64.1 ± 17.6 years, of whom 390 were men and 222 were women. The median overall RAR index was 4.61 (3.82–5.7), and its distribution is displayed in Figure 2. Using the RAR T1 group as the reference, an increase in RAR index was associated with a higher proportion of patients admitted to ICU, an increased likelihood of women patients, older age, lower body weight, reduced SBP and DBP, higher levels of RDW, creatinine, and BUN, and lower levels of albumin and platelets. The SOFA and SAPSII scores were worse, and the prevalence of diabetes and peripheral vascular disease was higher. The proportion of patients with cerebrovascular disease was lower.

**Table 1:** Comparisons of the baseline characteristics categorized by the RAR index.

Variables	Total overall, *N* = 612	RAR tertile 1 (*N* = 139)	RAR tertile 2 (*N* = 150)	RAR tertile 3 (*n* = 323)	*P-*value
RAR index	4.61 (3.82–5.7)	3.4 (3.16–3.55)	4.15 (3.93–4.32)	5.6 (4.98–6.61)	<0.001
Age, years	64.1 ± 17.6	56.8 ± 17.6	65.3 ± 17.9	66.6 ± 16.6	<0.001
Sex, male, *n* (%)	390 (63.73)	106 (76.26)	93 (62.00)	191 (59.13)	0.002
Weight, kg	86.4 ± 23.5	92.2 ± 21.5	86.2 ± 22.2	84.5 ± 25.0	0.006
Race, *n* (%)					0.703
White	329 (53.8)	71 (51.08)	84 (56.00)	174 (53.87)	
Other	283 (46.2)	68 (48.92)	66 (44.00)	149 (46.13)	
SBP, mmHg	114.7 ± 15.8	117.7 ± 16.3	117.8 ± 16.0	112.0 ± 15.1	<0.001
DBP, mmHg	64.0 ± 11.2	68.7 ± 11.1	65.0 ± 11.6	61.5 ± 10.3	<0.001
Heart rate, beats/min	84.93 ± 19.13	79.1 ± 16.9	81.7 ± 19.4	88.9 ± 19.0	<0.001
Respiratory rate, times/min	21.3 ± 4.7	21.1 ± 4.5	21.2 ± 4.3	21.5 ± 5.0	0.608
SpO_2_, %	97.7 (96.0–99.1)	97.9 (96.3–99.3)	98.0 (96.1–99.4)	97.5 (95.8–98.9)	0.012
Comorbidities, *n* (%)					
Cardiovascular disease	151 (24.67)	36 (25.9)	39 (26.0)	76 (23.53)	0.786
Chronic pulmonary disease	165 (26.96)	30 (21.58)	45 (30.00)	90 (27.86)	0.237
Diabetes	190 (31.03)	23 (16.55)	48 (32.00)	119 (36.84)	<0.001
Cerebrovascular disease	90 (14.71)	25 (17.99)	29 (19.33)	36 (11.15)	0.030
Peripheral vascular disease	87 (14.22)	11 (7.91)	18 (12.00)	58 (17.96)	0.012
Congestive heart failure	231 (37.75)	46 (33.09)	61 (40.67)	124 (38.39)	0.390
Norepinephrine use, *n* (%)	405 (66.18)	86 (61.87)	94 (62.67)	225 (69.66)	0.155
Scoring systems					
SOFA	8.7 ± 4.1	7.3 ± 3.8	7.8 ± 3.7	9.5 ± 4.1	<0.001
SAPSII	49.1 ± 17.0	40.8 ± 17.0	46.8 ± 15.5	53.8 ± 16.1	<0.001
Laboratory tests					
ALT, IU/L	72 (32–221)	98 (40–303)	76 (34–201)	61 (28–217)	0.038
AST, IU/L	118 (49–357)	118 (50–374)	118 (51–275)	118 (47–367)	0.910
ALP, IU/L	86 (62–124)	75 (61–97)	81 (62–113)	95 (61–134)	<0.001
RDW, %	14.6 (13.6–16.0)	13.2 (12.6–13.8)	14.2 (13.5–15.0)	15.6 (14.5–17.3)	<0.001
Albumin, g/dl	3.3 (2.7–3.7)	4.0 (3.8–4.2)	3.4 (3.2–3.7)	2.8 (2.4–3.1)	<0.001
WBC, 10^9^/L	13.4 (9.4–17.8)	13.7 (11.1–18.4)	12.9 (9.6–16.7)	13.3 (8.8–18.0)	0.181
RBC, 10^12^/L	3.8 (3.2–4.5)	4.5 (4.0–4.8)	4.0 (3.5–4.5)	3.4 (3.0–4.1)	<0.001
Platelets, 10^9^/L	188 (141–249)	203 (172–247)	182 (146–232)	181 (122–260)	0.010
Hemoglobin, g/dl	11.6 ± 2.4	13.6 ± 1.9	12.3 ± 1.9	10.4 ± 2.1	<0.001
Anion gap, mmol/L	16.7 (14.3–19.7)	16.3 (14–18.8)	16.5 (14.5–18.8)	17 (14.3–20.2)	0.223
Potassium, mg/dl	4.2 (3.9–4.7)	4.17 (3.9–4.7)	4.2 (3.9–4.5)	4.2 (3.8–4.8)	0.523
Sodium, mg/dl	139.0 (136.4–141.7)	139.6 (137.8–142.0)	138.4 (136.2–141.4)	138.8 (136.0–141.7)	0.031
Creatinine, mg/dl	1.4 (0.9–2.2)	1.1 (0.9–1.6)	1.3 (0.9–2.0)	1.7 (1.0–2.6)	<0.001
BUN, mg/dl	25.2 (17.2–40.6)	20.0 (15.0–26.5)	24.3 (17.5–37.0)	30.3 (18.5–52.3)	<0.001
Glucose, mg/dl	186.0 ± 83.0	184.2 ± 77.2	185.1 ± 76.4	187.3 ± 8.4	0.922
INR	1.3 (1.2–1.71)	1.16 (1.1–1.3)	1.2 (1.1–1.5)	1.5 (1.2–1.9)	<0.001
30-day mortality, *n* (%)	318 (51.96)	50 (35.97)	78 (52)	190 (58.82)	<0.001
180-day mortality, *n* (%)	362 (59.15)	55 (39.57)	90 (60.00)	217 (67.18)	<0.001

RAR, ratio of RDW to albumin; SBP systolic blood pressure; DBP, diastolic blood pressure; SOFA, Sequential Organ Failure Assessment; SAPSII, Simplified Acute Physiology Score II; ALT, alanine aminotransferase; AST, aspartate aminotransferase; ALP, alkaline phosphatase; RDW, red blood cell distribution width; WBC, white blood cell; RBC, red blood cell; BUN, blood urea nitrogen; INR, international normalized ratio.

### Associations between the RAR index and mortality risk in post-CA patients

When considering the RAR index as a continuous variable, Cox regression analysis revealed that an elevated RAR index was significantly associated with higher 30- and 180-day mortality in both the unadjusted model (30-day: HR: 1.14; 95% CI: 1.08–1.21; 180-day: HR: 1.16; 95% CI: 1.10–1.22) and the fully adjusted model (30-day: HR: 1.08; 95% CI: 1.01–1.15; 180-day: HR: 1.09; 95% CI: 1.03–1.16). When analyzing the RAR index as a categorized variable, Cox proportional hazards analysis revealed that the highest tertile (T3) of the RAR index was also significantly correlated with increased 180-day mortality in both the unadjusted model (HR: 2.07; 95% CI: 1.57–2.79) and the adjusted model (Model 1: HR: 2.00; 95% CI: 1.48–2.72; Model 2: HR: 1.74; 95% CI: 1.26–2.42) (Table 2). Similar results were also observed between the highest tertile (T3) of the RAR index and 30-day all-cause mortality (unadjusted: HR: 1.90; 95% CI: 1.39–2.60; Model 1: HR: 1.83; 95% CI: 1.33–2.52; Model 2: HR: 1.60; 95% CI: 1.13–2.25).

**Table 2:** Cox proportional hazards analysis of 30- and 180-day all-cause mortality in post-CA patients.

Outcomes	Groups	Non-adjusted		Model 1^a^		Model 2^b^	
		HR (95% CI)	*P*	HR (95% CI)	*P*	HR (95% CI)	*P*
30-day mortality	Continuous	1.14 (1.08–1.21)	0.0001	1.15 (1.08–1.22)	0.0001	1.08 (1.01–1.15)	0.0016
	T1 (<3.7; *N* = 135)	1		1		1	
	T2 (3.7–4.5; *N* = 155)	1.58 (1.10–2.25)	0.012	1.55 (1.08–2.22)	0.018	1.45 (1.00–2.09)	0.045
	T3 (>4.5; *N* = 322)	1.90 (1.39–2.60)	0.0001	1.83 (1.33–2.52)	0.0001	1.60 (1.13–2.25)	0.008
180-day mortality	Continuous	1.16 (1.10–1.22)	0.0001	1.16 (1.10–1.23)	0.0001	1.09 (1.03–1.16)	0.003
	T1 (<3.7; *N* = 135)	1		1		1	
	T2 (3.7–4.5; *N* = 155)	1.71 (1.23–2.40)	0.002	1.69 (1.20–2.37)	0.003	1.59 (1.12–2.25)	0.009
	T3 (>4.5; *N* = 322)	2.07 (1.54–2.79)	0.0001	2.00 (1.48–2.72)	0.0001	1.74 (1.26–2.42)	0.001

Models 1 and 2 were derived from Cox proportional hazards regression models.

^a^ Model 1 covariates were adjusted for age, race, weight, and sex.

^b^ Model 2 covariates were adjusted for age, race, weight, sex, SOFA score, SAPSII score, cardiovascular disease, chronic pulmonary disease, diabetes, cerebrovascular disease, peripheral vascular disease, norepinephrine use, creatinine, SBP, DBP, ALT, ALP, and AST.

**Figure 3:**
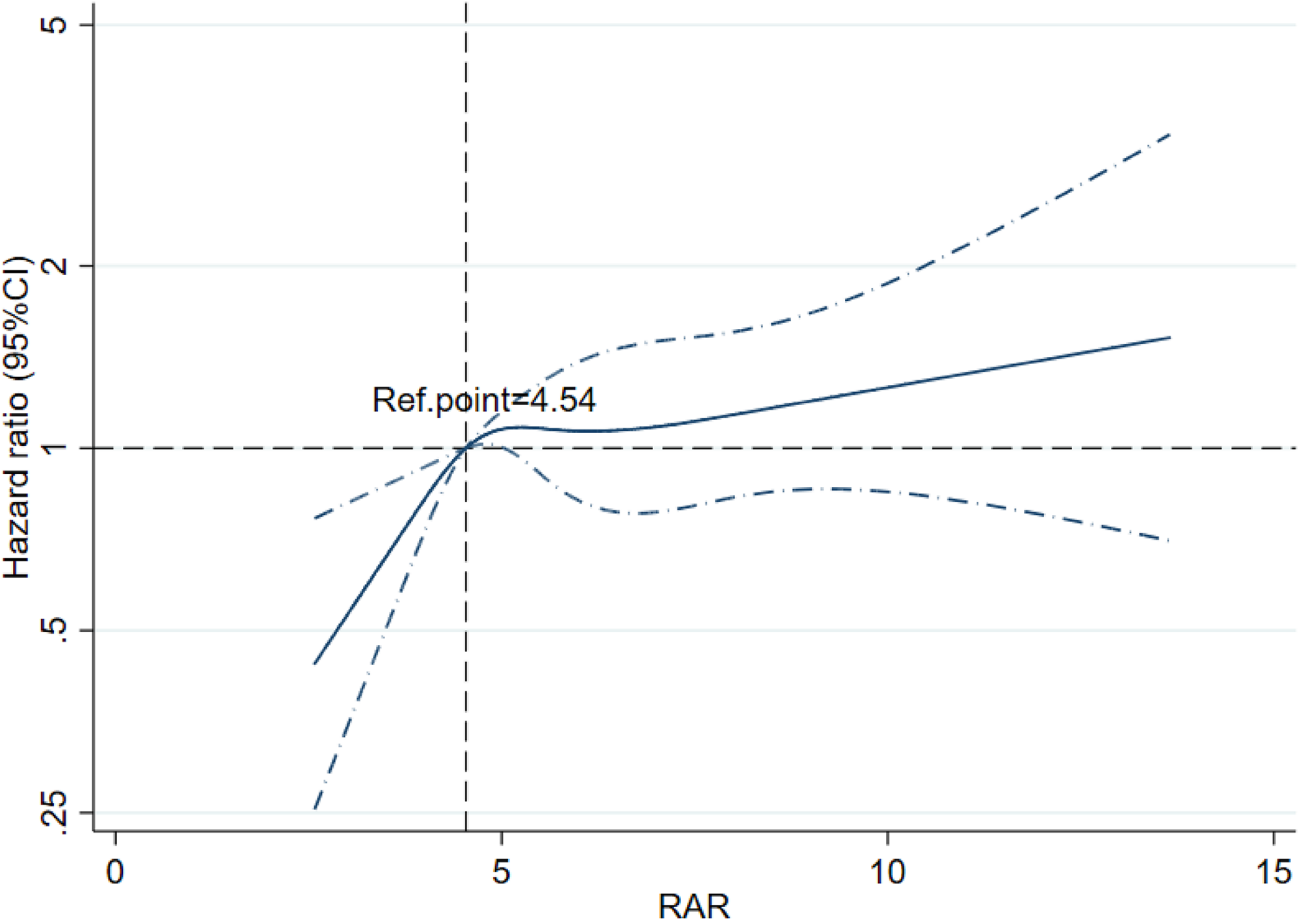
Restricted cubic spline regression analysis of the RAR index for 180-day mortality among post-CA patients.

**Figure 4:**
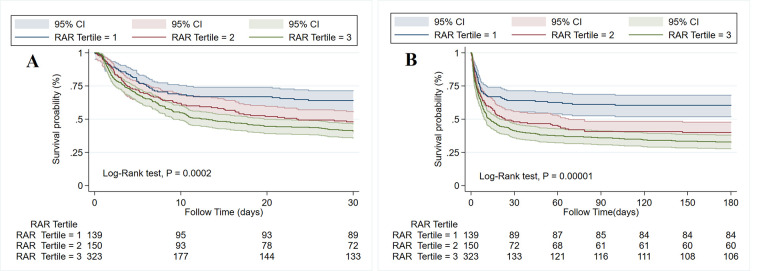
Kaplan–Meier survival curve of the cumulative survival rate among three tertiles of the RAR index within 30-day **(A)** and 180-day **(B)** follow-ups.

RCS analysis indicated a non-linear relationship between the RAR index and 180-day mortality in patients with CA (Figure 3). The risk of mortality increased with increasing RAR levels, and its HR was always >1 when the RAR level exceeded 4.54. The cumulative 30-day and 180-day mortality across RAR index groups is shown in Kaplan‒Meier analysis (30-day: T1: 50, 35.97%; T2: 78, 52%; T3: 190, 58.82%; 180-day: T1: 55, 39.57%; T2: 90, 60%; T3: 217, 67.18%) (see Figure 4). The analysis revealed that the cumulative incidence of 30- and 180-day death increased with elevating quartiles of the RAR index (log-rank test, *p* < 0.001).

### ROC curve analysis of the RAR index

**Figure 5:**
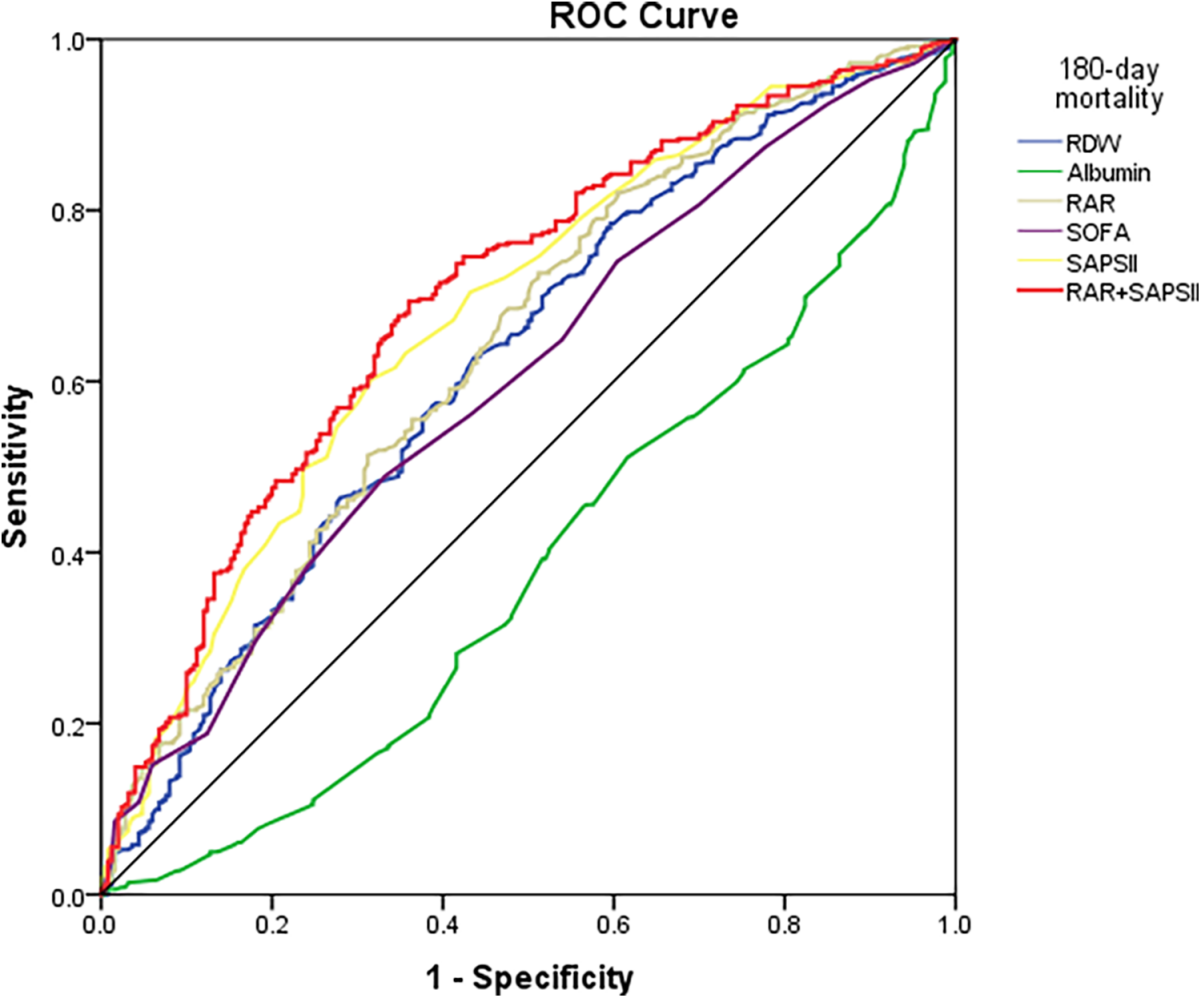
ROC curves predicting 180-day mortality in CA patients.

ROC curve analyses were conducted for the RAR index, RDW, albumin, SOFA score, SAPSII score, and SAPSII combined RAR to predict 180-day mortality in patients with CA (Figure 5). It was observed that the RAR index [0.640 (0.596–0.685)] outperformed the RDW [0.623 (0.577–0.668)], albumin [0.384 (0.339–0.429)], and SOFA score [0.601 (0.556–0.646)] in predicting all-cause 180-day mortality, both with *p* < 0.001. In addition, the RAR index showed no significant superiority over the SAPSII score [0.677 (0.634–0.720)] for CA patients. Further analysis of the combined RAR index and SAPSII score for prognostic assessment in CA patients showed a better predictive capability [0.698 (0.655–0.740)] compared to the RAR index and SAPSII score alone.

### Subgroup analyses

After adjusting for all covariates, we conducted subgroup analyses based on demographics and several comorbidities. As shown in Table 3, the positive association between the RAR index and 180-day mortality remained generally consistent across subgroups. An interaction was found in the strata of race (*p* for interaction = 0.025), chronic pulmonary disease (*p* for interaction = 0.005), and cerebrovascular disease (*p* for interaction = 0.010). Patients with higher RAR levels were related to higher mortality among white patients, those with chronic pulmonary disease, or those without cerebrovascular disease.

**Table 3:** Subgroup analyses of the association between the RAR index and 180-day all-cause mortality.

Variables	No. of patients	RAR index			*P* for interaction
		T1 (3.7)	T2 (3.7–4.5)	T3(>4.5)	
Sex					0.404
Male	390	1.0	1.51 (0.99–2.32)	2.04 (1.37–3.04)	
Female	222	1.0	1.39 (0.73–2.67)	1.06 (0.58–1.95)	
Age					0.116
<60	219	1.0	1.54 (0.90–2.62)	1.51 (0.92–2.45)	
≥60	393	1.0	1.75 (1.06–2.88)	2.15 (1.33–3.46)	
Race					0.025
White	329	1.0	1.75 (1.06–2.88)	2.15 (1.33–3.46)	
Other	283	1.0	1.54 (0.90–2.62)	1.51 (0.92–22.45)	
Cardiovascular disease					0.531
No	461	1.0	1.55 (1.04–2.30)	1.71 (1.18–2.48)	
Yes	151	1.0	1.64 (0.76–3.53)	1.77 (0.84–3.75)	
Diabetes					0.721
No	422	1.0	1.63 (1.10–2.43)	1.94 (1.34–2.81)	
Yes	190	1.0	1.54 (0.66–3.56)	1.41 (0.65–3.07)	
Chronic pulmonary disease					0.210
No	447	1.0	1.44 (0.98–2.13)	1.52 (1.06–2.18)	
Yes	165	1.0	3.72 (1.48–9.32)	3.33 (1.30–8.53)	
Cerebrovascular disease					0.005
No	522	1.0	1.42 (0.95–2.13)	1.90 (1.31–2.76)	
Yes	90	1.0	3.63 (1.63–8.09)	1.31 (0.56–3.05)	
Peripheral vascular disease					0.010
No	525	1.0	1.42 (0.98–2.05)	1.80 (1.28–2.53)	
Yes	87	1.0	5.59 (1.68–18.62)	1.13 (0.33–3.83)	
Norepinephrine use					0.781
No	207	1.0	1.48 (0.77–2.85)	1.24 (0.65–2.37)	
Yes	405	1.0	1.61 (1.05–2.46)	1.84 (1.24–2.72)	

HRs (95% CIs) were derived from Cox regression models. Covariates were adjusted as in model 2 (Table 2).

## Discussion

Recently, mortality in CA patients has been gradually found to be associated with an anion gap, albumin-corrected anion gap (21), INR (22), and the stress hyperglycemia ratio (23), which reflects tissue hypoxia, abnormalities in the coagulation–fibrinolysis system, and electrolyte disturbances. However, few studies have explored the predictive power of inflammatory indicators in determining mortality among CA patients. To our knowledge, this study is the first to underscore that elevated RAR levels, a novel inflammatory biomarker, are associated with higher 30- and 180-day all-cause mortality in post-CA patients admitted to the ICU, both as a continuous and nominal variable. First, we used RCS analysis to investigate the potential correlation between the RAR index and 180-day prognosis. There was a corresponding increase in all-cause mortality with an increased RAR index. When RAR was >4.54, the risk of all-cause death was increased. The K‒M survival curve and Cox regression analysis further indicated that the 30- and 180-day mortality risks were noticeably higher in CA patients with an elevated RAR index, which was still robust in the subgroup analyses. Therefore, our findings could help clinicians initially predict the prognosis of CA patients within 180 days using an easily accessible index.

Post-CA patients suffer from systemic ischemia/reperfusion injury (24), which triggers the activation of systemic inflammation, contributes to hemodynamic instability, and exacerbates injury to the brain and myocardium (25). Previous studies have shown that high levels of inflammatory response in CA patients are associated with higher mortality and/or worse neurologic consequences (16, 17). It is significant for clinicians to identify effective biomarkers that can reflect systemic inflammation and predict the mortality of patients after CA.

RDW measures the degree of heterogeneity in RBC volume, as detected by a blood analysis, and a high RDW reflects severe erythrocyte homeostasis deregulation caused by various metabolic abnormalities, such as inflammation (26). Previous studies have demonstrated that high RDW is associated with all-cause mortality in patients with cardiovascular disease, diabetes, cancer, liver and kidney failure, and other diseases (27). Similarly, albumin levels are usually utilized to analyze the nutritional status of patients (28) and have now been proposed as an inflammation biomarker of prognosis in critically ill patients (29). It has been shown that the synthesis rate of albumin is negatively associated with inflammatory activity (30), and low albumin levels in the early phase play a pro-inflammatory role and are positively correlated with hospitalization mortality of patients after CA (31).

Currently, the RAR index has emerged as a rapid, reproducible, and easily accessible composite marker that combines both nutritional status (ALB) and inflammatory status (RDW), and it may be a better tool to reflect the inflammatory response and identify high-risk patients compared to other single-identified markers (RDW and albumin). Xu et al. revealed that the RAR index is a potential prognostic indicator for sepsis patients, and a higher RAR index indicates worse clinical prognosis (19). Li and Xu reported that a high RAR index is a potential marker of increased mortality in AMI patients (14). However, no studies have explored the relationship between the RAR index and CA patients. Remarkably, this study indicated that after adjusting for age, race, weight, sex, SOFA score, SAPSII score, cardiovascular disease, chronic pulmonary disease, diabetes, cerebrovascular disease, peripheral vascular disease, norepinephrine use, creatinine, SBP, DBP, ALT, ALP, and AST, the RAR index was positively correlated with increased 180-day mortality in patients with CA. It is suggested that a higher RAR level is associated with worse prognosis in post-CA patients.

In our study, the RAR index is an independent predictor of 180-day mortality in CA patients. It showed superior predictive value compared to standalone markers, such as RDW, albumin, or SOFA scores. This indicates that the RAR index may be an available supplementary measure to clinical decision-making for prognostic assessment in CA patients. For ICU patients, particularly those with CA, routine monitoring of RAR may be advisable to remind clinicians to quickly identify high-risk patients. In addition, combining RAR with the SAPSII score may improve predictive significance.

The mechanisms by which the RAR level influences 180-day mortality in CA patients remain elusive but are hypothetically related to chronic inflammation and nutritional deficiencies. Cardiac arrest-induced inflammation could lead to increased RDW elevation and decreased albumin levels, which causes a significant increase in RAR. However, our study was unable to assess the indirect effect of inflammatory markers [such as C-reactive protein, hypersensitive C-reactive protein, erythrocyte sedimentation rate (ESR), and ferritin] on the association between the RAR level and the likelihood of 180-day mortality through mediation analysis because of the high number of missing values. Gaining insight into these underlying mechanisms is propitious to ascertain potential interventions that focus on regulating the inflammatory environment and improving outcomes in patients with CA.

Our study indicated that the cutoff value of the RAR index for predicting 180-day mortality is 4.54. The RAR index combines both nutritional status (ALB) and inflammatory status (RDW); the elevation in RAR often stems from higher RDW and/or lower albumin levels. We hypothesized that when the RAR index exceeds 4.54, post-CA patients may have developed severe inflammation and/or poor nutritional status, which is difficult to reverse. Several previous reports have also shown a similar finding. A study conducted on patients with coronary heart disease and diabetes mellitus demonstrated that individuals with a RAR cutoff value >4.26 exhibited significantly higher 1-year mortality compared to those with a RAR cutoff value <4.26 [area under curve (AUC) 0.68] (32). Similarly, another study focusing on patients with sepsis and atrial fibrillation revealed that the optimal cutoff value of RAR for predicting in-hospital mortality was 4.882 (15).

Furthermore, subgroup analyses showed a significant interaction among patients with chronic pulmonary disease. This may be attributed to hypoxic conditions and inflammation responses, as acute hypoxia and inflammatory responses could promote an increase in serum erythropoietin and lead to greater variability in red blood cells, which is reflected by elevated RDW (33). However, enlarged red blood cells in the circulatory system would lead to inferior capabilities of transport oxygen, which likely exacerbates hypoxia (34).

Nevertheless, our study has some drawbacks. First, we used a retrospective study design, and the selection bias and confounding bias of the retrospective study itself cannot be ignored, although we adjusted for potential confounding confounders and performed subgroup analyses. Future prospective cohort studies are needed to validate our outcomes. Second, we tested RAR only at the time of ICU admission without dynamically supervising the RAR index, which may change over time or in response to the condition of the patients after CA. Third, due to the limitation of the database, some variables, including cardiac function classification, echocardiography results, care conditions out of hospital, and therapy (potential confounders), were unavailable, which might impact our results. Finally, the potential mechanism between a higher RAR index and elevated mortality in post-CA patients remains unclear, warranting further research. In addition, since ischemia–reperfusion injury and inflammatory responses play key roles in the pathophysiology of cardiac arrest, it may be worth exploring the association between other inflammatory or hypoxic indicators and mortality among CA patients.

## Conclusion

Our study identified the RAR index as an independent prognostic marker for post-CA patients, and an increased RAR index was associated with higher 180-day mortality in post-CA patients. The RAR index is expected to be an available and effective prognostic evaluation indicator in post-CA patients.

## Data Availability

Publicly available datasets were analyzed in this study. This data can be found here: https://physionet.org/content/mimiciv/2.0/.
